# Integrative analysis of recurrence related gene signature and STC1 in colorectal cancer proliferation and metastasis

**DOI:** 10.7150/jca.102605

**Published:** 2024-10-28

**Authors:** Chao Xu, ShuYuan Li, HongYuan Chen, LiangJie Chi, XiangYu Wang, Muzhen He, Qingshui Wang, Xiuli Zhang, Yao Lin, FangQin Xue

**Affiliations:** 1 Department of Gastrointestinal Surgery, Shengli Clinical Medical College of Fujian Medical University, Fujian Provincial Hospital, Fuzhou University Affiliated Provincial Hospital, No. 134 Dongjie, Fuzhou, China.; 2 Department of Radiology, Shengli Clinical Medical College of Fujian Medical University, Fujian Provincial Hospital, Fuzhou University Affiliated Provincial Hospital, No. 134 Dongjie, Fuzhou, China.; 3 Fujian-Macao Science and Technology Cooperation Base of Traditional Chinese Medicine-Oriented Chronic Disease Prevention and Treatment, College of Integrative Medicine, Fujian University of Traditional Chinese Medicine, Fuzhou, China.

**Keywords:** Colorectal cancer, Recurrence, Recurrence risk gene signature, STC1, Prognostic biomarkers

## Abstract

Colorectal cancer remains a formidable global health challenge, characterized by high recurrence rates and poor prognosis. This study introduces a novel Recurrence Related Gene Signature (RRGS), designed to predict therapy response and enhance prognostic accuracy in colorectal cancer. Through analysis of the GSE17536 cohort, we identified 79 differentially expressed genes (DEGs) between recurrent and non-recurrent cases, comprising 54 upregulated and 25 downregulated genes. Pathway analysis revealed that upregulated genes were enriched in cancer progression-related pathways, while downregulated genes were associated with immune-related processes. Leveraging these findings, we developed the RRGS using LASSO regression, resulting in an innovative 11-gene model with robust diagnostic and prognostic capabilities. Notably, the RRGS demonstrated significant predictive value for both overall survival and disease-free survival across multiple datasets, with higher RRGS scores correlating with advanced tumor stages and poorer outcomes, particularly in post-chemotherapy patients. This predictive power highlights the RRGS's potential in guiding personalized treatment strategies. Furthermore, we identified STC1 as a critical component of the RRGS, playing a significant role in tumor progression and immune evasion. Through rigorous *in vitro* and *in vivo* experiments we confirmed that STC1 knockdown substantially reduced cell proliferation and metastasis, emphasizing its potential as a therapeutic target. This comprehensive study not only elucidates the molecular mechanisms driving colorectal cancer recurrence but also introduces a powerful tool for enhancing prognostic accuracy and personalizing therapeutic interventions.

## Introduction

Colorectal cancer is among the most prevalent malignancies globally and remains a leading cause of cancer-related morbidity and mortality^1^-^3^. Despite significant advancements in screening and therapeutic interventions, a substantial number of patients experience disease recurrence, posing a major challenge in clinical management^4^-^6^. Recurrence is often associated with a poor prognosis and limited treatment options, highlighting the urgent need for improved strategies to predict and prevent this outcome^7^.

Recurrence of colorectal cancer can manifest as local recurrence or distant metastases, both of which significantly impact patient survival and quality of life^8^, ^9^. Current prognostic models primarily rely on clinical and pathological features, which may not fully capture the underlying biological complexity driving recurrence^10^-^12^. This underscores the critical need to identify reliable biomarkers that can predict recurrence risk and inform personalized treatment strategies.

Biomarkers for colorectal cancer recurrence have the potential to revolutionize patient management by enabling early intervention and tailored therapeutic approaches^13^. Molecular markers, in particular, can provide valuable insights into the genetic and epigenetic alterations associated with tumor progression and recurrence. Identifying such biomarkers could facilitate the development of targeted therapies and enhance surveillance strategies, ultimately improving patient outcomes.

Stanniocalcin-1 (STC1) is a secreted glycoprotein hormone originally identified in fish, where it regulates calcium and phosphate homeostasis. In humans, STC1 is widely expressed across various tissues and plays roles in multiple physiological and pathological processes, including cancer development^14^. In tumorigenesis, STC1 is often overexpressed in several cancer types such as ovarian, and esophageal cancers^15^, ^16^. Elevated STC1 levels promote cancer cell proliferation, migration, invasion, and angiogenesis, thereby facilitating tumor growth and metastasis. Additionally, high STC1 expression is associated with poor prognosis and reduced survival rates in cancer patients, making it a potential biomarker for cancer progression and a target for therapeutic intervention^17^. STC1 is also involved in the regulation of key signaling pathways, including PI3K/Akt and JNK/AP1, which are critical for cancer cell survival and adaptation to hypoxic conditions within the tumor microenvironment^15^.

In this study, we aim to explore the genetic landscape associated with colorectal cancer recurrence, with a particular focus on the role of STC1. By analyzing differentially expressed genes in recurrent versus non-recurrent colorectal cancer tissues, we seek to investigate the contribution of STC1 to cancer progression and its potential as a prognostic indicator and therapeutic target. Our findings may contribute to a deeper understanding of the molecular mechanisms underlying colorectal cancer recurrence and support the advancement of precision oncology in this field.

## Methods

### Data acquisition and preprocessing

GSE17536^18^ and GSE40967^19^ cohorts were obtained from the GEO database. The Affy package in R was employed to normalize and preprocess the microarray data using the Robust Multi-array Average (RMA) method. Gene expression data of colorectal cancer, quantified as fragments per kilobase million (FPKM), along with corresponding clinical information, were retrieved from The Cancer Genome Atlas (TCGA) via the UCSC XENA platform (https://xenabrowser.net/datapages/). These FPKM values were subsequently converted to transcripts per kilobase million (TPM) for downstream analysis.

### Cohort information

The GSE17536 cohort consists of 177 colorectal cancer patients with clinical follow-up data, including 36 patients who experienced recurrence, 109 patients who did not, and 32 patients for whom recurrence information is missing. The GSE40967 cohort includes 585 colorectal cancer patients, with 240 having undergone chemotherapy. The TCGA cohort provides information on 459 colorectal cancer patients.

### Differential expression analysis of genes between recurrence and without recurrence in colorectal cancer

We utilized the 'limma' package in R to identify differentially expressed genes (DEGs) in the GSE17536 cohort, comparing patients with and without colorectal cancer recurrence. Genes with a |log2FC| ≥ 0.585 and a p-value < 0.01 were considered significant DEGs.

### Enrichment analysis

To investigate the potential biological processes and pathways in which these differentially expressed genes may participate, we conducted enrichment analyses using Kyoto Encyclopedia of Genes and Genomes (KEGG)^20^, Gene Ontology (GO) gene sets^21^, and Hallmark gene sets^22^ pathways. A threshold of an adjusted p-value less than 0.05 was applied to determine the significance of pathways or processes.

### Least Absolute Shrinkage and Selection Operator regression (LASSO)

We employed LASSO logistic regression to filter DEGs and construct a diagnostic model for colorectal cancer recurrence, known as the RRGS model. LASSO is a powerful statistical method that uses L1 regularization to penalize the absolute magnitude of the coefficients of the regression variables. This regularization technique encourages sparsity, effectively selecting a subset of genes by shrinking some of their coefficients to zero, which helps avoid overfitting and enhances model interpretability^23^. The RRGS formula was derived through the LASSO analysis using the "glmnet" package in R, which fits generalized linear models via penalized maximum likelihood. The RRGS is mathematically expressed as: RRGS = (A₁ × E₁) + (A₂ × E₂) + ... + (Aₙ × Eₙ). Here, A₁, A₂, ..., Aₙ represent the coefficients for genes 1 to n, while E₁, E₂, ..., Eₙ denote the expression levels of these genes. This formula calculates a score reflecting the likelihood of colorectal cancer recurrence, facilitating early intervention strategies. The diagnostic efficacy of the gene signatures and the resulting model was evaluated using receiver operating characteristic (ROC) curves.

### Gene mutation analysis

The GSCA database (https://guolab.wchscu.cn/GSCA/#/) functions as a cancer genomics portal tailored for gene set cancer analysis^24^. In this study, it was employed to investigate the relationship between the expression of genes and single nucleotide variations (SNVs) in colorectal cancer.

### Immune microenvironment analysis

To evaluate immune infiltration in colorectal cancer, we applied the ESTIMATE, CIBERSORT, and XCELL algorithms. Subsequently, we examined the correlation between RRGS and different immune cell types.

### Immunohistochemistry analysis using the human protein atlas (THPA)

Immunohistochemistry images were sourced from THPA (www.proteinatlas.org/), a publicly available database that houses over 500,000 images of immunohistochemically stained tissues and cells. The data on STC1 expression levels in colorectal cancer tissues were also retrieved from THPA.

### Cell culture

The human colorectal cancer cell lines HCT-116 and DLD1 were obtained from the American Type Culture Collection (ATCC). The cells were cultured in Dulbecco's Modified Eagle Medium (DMEM), enriched with 10% fetal bovine serum (FBS) and 1% penicillin-streptomycin, and maintained at 37°C in a humidified atmosphere with 5% CO_2_.

### Quantitative Real-Time PCR (qRT-PCR)

Total RNA was extracted from the cells using TRIzol reagent (Invitrogen) and reverse-transcribed into cDNA with the PrimeScript RT reagent Kit (TaKaRa, Japan). QRT-PCR was performed with SYBR Premix Ex Taq (TaKaRa), using GAPDH as an internal control. Relative expression levels were calculated using the 2^-ΔΔCt^ method. The primers for STC1 were as follows: forward 5'-ACAGCAAGCTGAATGTGTGC-3' and reverse 5'-CAGGCTTCGGACAAGTCTGT-3'. For GAPDH, the forward primer was 5'-GGTGAAGGTCGGAGTCAAC-3', and the reverse primer was 5'-CAAATGAGCCCCAGCCTTC-3'.

### Cell proliferation assay

Cell proliferation was assessed using the Cell Counting Kit-8 (CCK-8) assay. Briefly, cells were seeded into 96-well plates at a density of 2 × 10³ cells per well. At 0, 24, 48, and 72 hours after seeding, CCK-8 solution was added to each well and incubated for 2 hours at 37°C. Absorbance was measured at 450 nm using a microplate reader.

### Wound healing assay

Cells were allowed to reach confluence in 6-well plates, and a scratch was made using a sterile 10-μL pipette tip. After washing with PBS, the cells were maintained in a serum-free medium. Images were captured at 0 and 48 hours using an inverted microscope, and the wound closure rate was calculated with ImageJ software.

### Transwell invasion assay

Cell invasion was evaluated using Matrigel-coated Transwell chambers (Corning, NY, USA). Briefly, 2 × 10⁴ cells were seeded into the upper chamber containing serum-free medium, while the lower chamber was filled with medium supplemented with 10% FBS. After 24 hours, non-invading cells were removed, and the invaded cells were fixed, stained with crystal violet, and counted using a microscope.

### Zebrafish xenograft model

We sourced zebrafish from Fuzhou Bio-Service Biotechnology Co. Ltd (Fuzhou, China). For the xenotransplantation procedure, we used GB100T-8P injection glass capillaries (Science Products GmbH, Germany) formed using FemtoJet 4i microinjectors (Eppendorf, Germany). HCT-116 cells were labeled with 5 μM of 1,1'-dioctadecyl-3,3,3',3'-tetramethylindocarbocyanine perchlorate (Dil; Meilun Biotechnology, China), a red-fluorescent lipophilic membrane dye. We injected approximately 200 labeled HCT-116 cells into either the center of the yolk sac or the ventral yolk cavity of each zebrafish larva using a microinjector, with ten larvae in each group. To assess tumor cell proliferation, we captured fluorescent images of all ten subjects in each group at 2 and 48 hours post-xenotransplantation. For evaluating metastatic spread, we imaged tail fluorescence at 2 and 24 hours post-transplantation. This imaging protocol allowed us to monitor both local tumor growth and distant metastasis in the zebrafish model. Experiments with zebrafish larvae under 5 days old do not require ethics committee approval. Our study followed the ARRIVE guidelines for reporting animal research.

### Statistical analysis

All experiments were performed in triplicate, and the data are presented as mean ± standard deviation (SD). Statistical analyses were conducted using GraphPad Prism 8.0 software. Differences between two groups were assessed with Student's t-test, while comparisons among multiple groups were analyzed using one-way ANOVA. A *p*-value of less than 0.05 was deemed statistically significant.

## Results

### Workflow of this study

**Figure 1:**
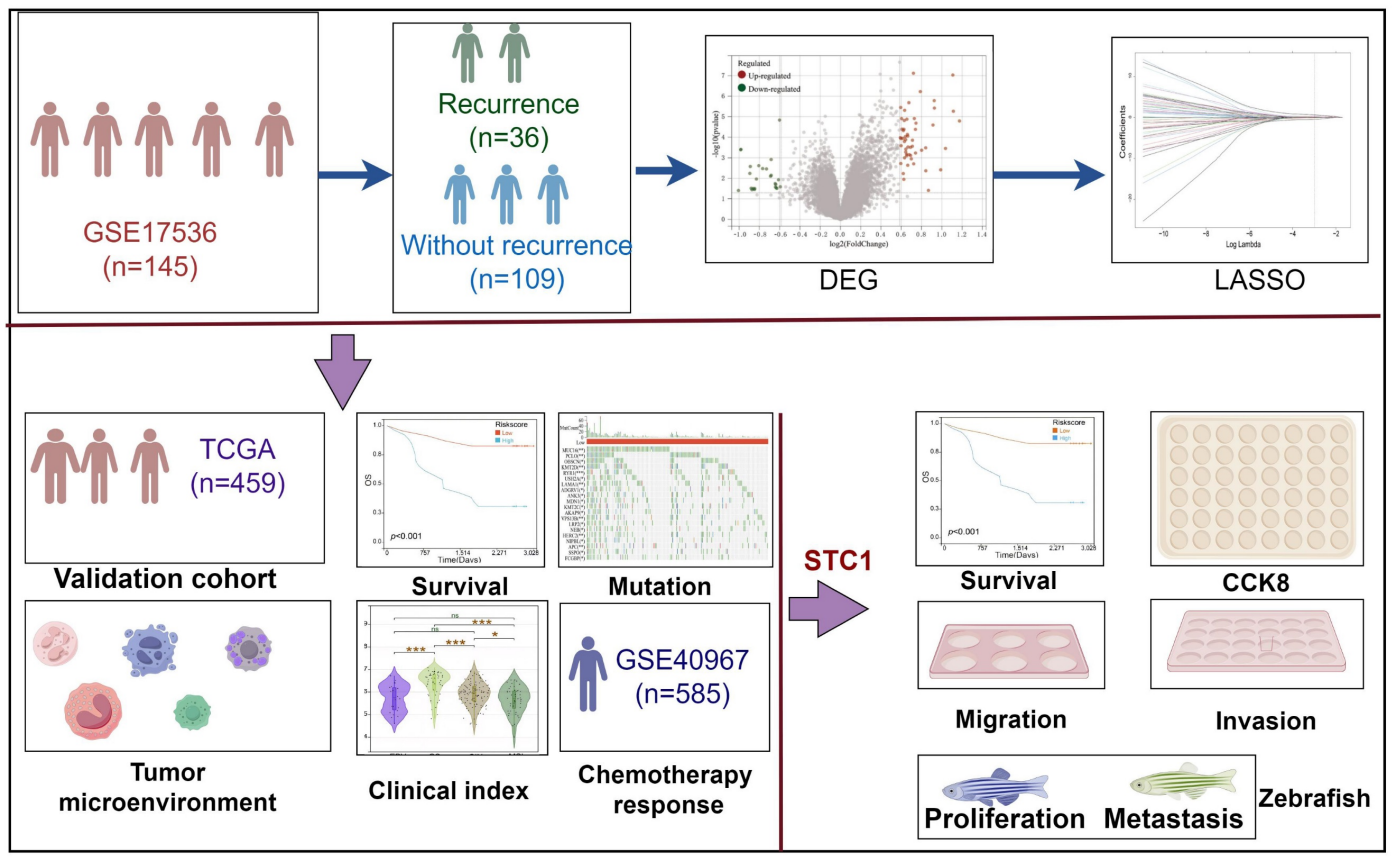
Flowchart for comprehensive analysis of prognostic model based on recurrence related genes in colorectal cancer.

In this study, we analyzed the differentially expressed genes between patients who experienced recurrence and those who did not in the GSE17536 cohort. Based on these genes, we developed a diagnostic model for colorectal cancer recurrence. The detailed workflow of this research is depicted in Figure 1.

### Identification of recurrence related gene in colorectal cancer

**Figure 2:**
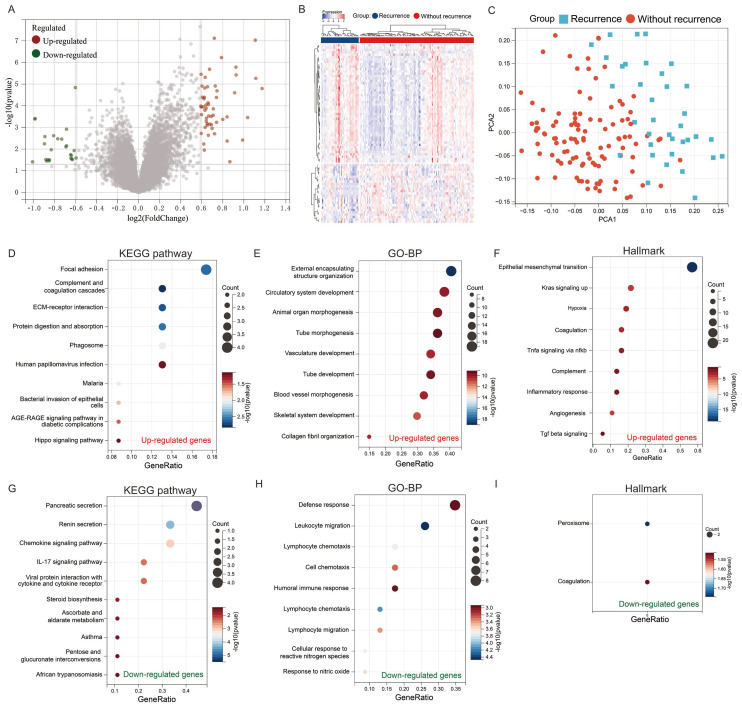
** Identification of key genes for recurrence in colorectal cancer.** (A) Volcano plot and (B) heatmap showing differentially expressed genes between recurrent and non-recurrent colorectal cancer patients in the GSE17536 dataset; (C) Principal Component Analysis (PCA); (D-F) Enrichment analysis of upregulated genes: (D) KEGG pathway, (E) GO-BP, and (F) Hallmark gene set; (G-I) Enrichment analysis of downregulated genes: (G) KEGG pathway, (H) GO-BP, and (I) Hallmark gene set.

To elucidate the genetic factors associated with colorectal cancer recurrence, we conducted a comprehensive analysis of the GSE17536 cohort, comprising tissue samples from 36 patients with recurrent colorectal cancer and 109 patients without recurrence. Our differential gene expression analysis revealed a distinct genetic profile between recurrent and non-recurrent colorectal cancer tissues. Specifically, we identified 54 significantly upregulated genes and 25 significantly downregulated genes in recurrent colorectal cancer tissues compared to non-recurrent tissues (Figures 2A & B). Principal component analysis (PCA) demonstrated that these 79 differentially expressed genes effectively discriminate between recurrent and non-recurrent colorectal cancer patients (Figure 2C).

To gain insight into the biological implications of these genetic alterations, we performed pathway enrichment analyses. KEGG pathway analysis of the upregulated genes revealed significant enrichment in pathways crucial for cancer progression, including focal adhesion, complement and coagulation cascades, ECM-receptor interaction, and protein digestion and absorption (Figure 2D). Complementary Gene Ontology Biological Process (GO-BP) analysis indicated that these genes are predominantly involved in processes essential for tumor development and metastasis, such as external encapsulating structure organization, circulatory system development, animal organ morphogenesis, and tube morphogenesis (Figure 2E). Hallmark analysis further corroborated these findings, showing enrichment in pathways associated with cancer progression, including epithelial-mesenchymal transition, Kras signaling activation, hypoxia, and coagulation (Figure 2F).

Conversely, the downregulated genes exhibited a different functional profile. KEGG pathway analysis of these genes highlighted enrichment in pancreatic secretion, renin secretion, chemokine signaling, and IL-17 signaling pathways (Figure 2G). GO-BP analysis revealed their involvement in immune-related processes, including defense response, leukocyte migration, lymphocyte chemotaxis, and cell chemotaxis (Figure 2H). Intriguingly, hallmark analysis indicated that these genes are enriched in peroxisome and coagulation pathways (Figure 2E).

### Construction of a diagnostic gene model for colorectal cancer recurrence using recurrence related genes

**Figure 3:**
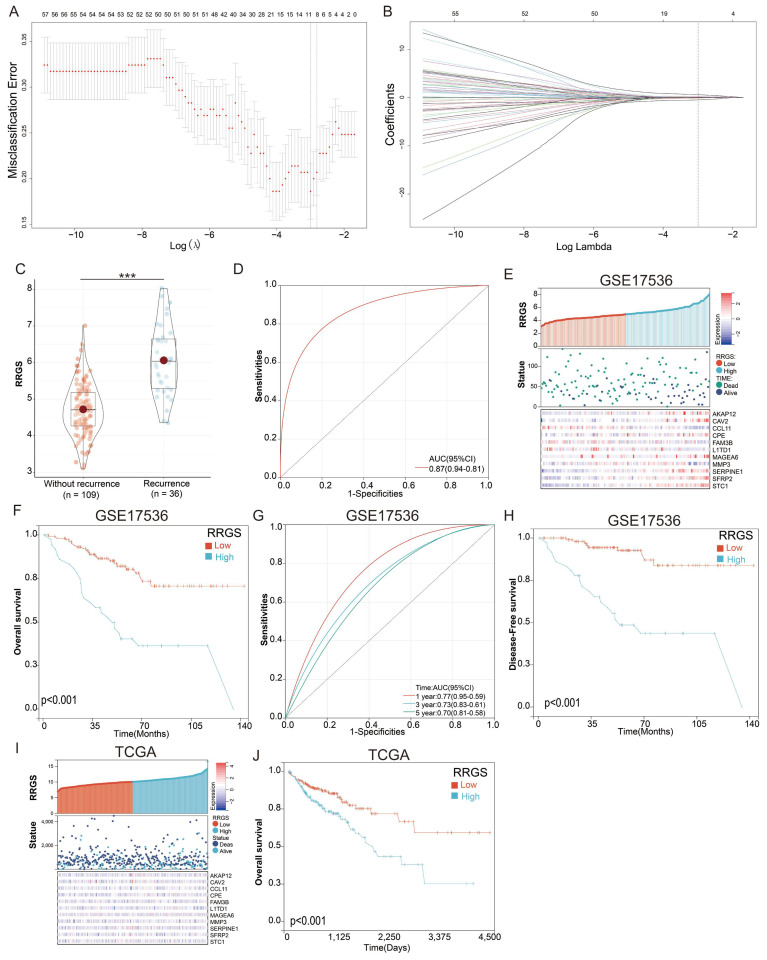
** Construction of a diagnostic model for colorectal cancer recurrence.** (A-B) Lasso regression for constructing the diagnostic model of colorectal cancer recurrence; (C) Differential analysis of RRGS values between recurrent and non-recurrent groups; (D) ROC analysis of RRGS for diagnosing colorectal cancer recurrence; (E) RRGS, survival status, and expression levels of the eleven genes in the GSE17536 cohort; (F) The impact of RRGS on patients' overall survival (OS) in the GSE17536 cohort; (G) Time-dependent ROC analysis of RRGS; (H) The impact of RRGS on patients' disease-free survival (DFS) in the GSE17536 cohort; (I) RRGS, survival status, and expression levels of the eleven genes in the TCGA cohort; (J) The impact of RRGS on patients' overall survival (OS) in the TCGA cohort.

We developed a diagnostic model using the LASSO regression based on the 79 differentially expressed genes identified in our previous research. To select the most relevant genes for the model, we applied LASSO regression with 10-fold cross-validation to determine the optimal penalty parameter (λ). This method effectively penalizes less significant variables, enhancing model performance and interpretability. From this analysis, 11 genes with non-zero coefficients were selected for inclusion in the final LASSO regression model (Figures 3A & B). The selection criteria focused on minimizing the cross-validated error while maintaining a parsimonious model to prevent overfitting. The final model's equation is as follows: RRGS = (0.023 × expression of AKAP12) + (0.325 × expression of CAV2) + (-0.448 × expression of CCL11) + (0.094 × expression of CPE) - (0.086 × expression of FAM3B) - (0.044 × expression of L1TD1) + (0.013 × expression of MAGEA6) - (0.045 × expression of MMP3) + (0.038 × expression of SERPINE1) + (0.116 × expression of SFRP2) + (0.643 × expression of STC1). Notably, among these genes, STC1 had the highest positive coefficient (0.643), indicating it was the most significant contributor to the model and suggesting a strong association with colorectal cancer recurrence.

Notably, the RRGS values were significantly higher in recurrent colorectal cancer tissues compared to non-recurrent tissues (Figure 3C), indicating the model's ability to distinguish between these groups. The diagnostic capability of the model was further validated using a ROC curve, which yielded an area under the curve (AUC) of 0.87 (Figure 3D), demonstrating strong predictive power.

In evaluating the prognostic significance of RRGS for colorectal cancer patients, we observed distinct expression levels of the model genes AKAP12, CAV2, CCL11, CPE, FAM3B, L1TD1, MAGEA6, MMP3, SERPINE1, SFRP2, and STC1, along with survival status and duration in RRGS-Low and RRGS-High subtypes (Figure 3E). Overall survival (OS) analysis indicated that patients classified as RRGS-High had significantly poorer survival outcomes compared to those in the RRGS-Low group (Figure 3F). Furthermore, ROC analysis demonstrated that the RRGS predictor achieved an AUC of 0.77, 0.73, and 0.70 for one-year, three-year, and five-year prognostication, respectively (Figure 3G). Similar trends were observed in disease-free survival (DFS) analysis, where the RRGS-High group experienced markedly shorter DFS compared to the RRGS-Low group (Figure 3H). Additional validation using the TCGA cohort confirmed these findings, showcasing gene expression and survival data across RRGS subtypes (Figure 3I). Consistently, OS analysis reinforced that the RRGS-High subtype was associated with significantly reduced overall survival relative to the RRGS-Low subtype (Figure 3J).

### Correlation between RRGS and higher tumor stage and EMT

**Figure 4:**
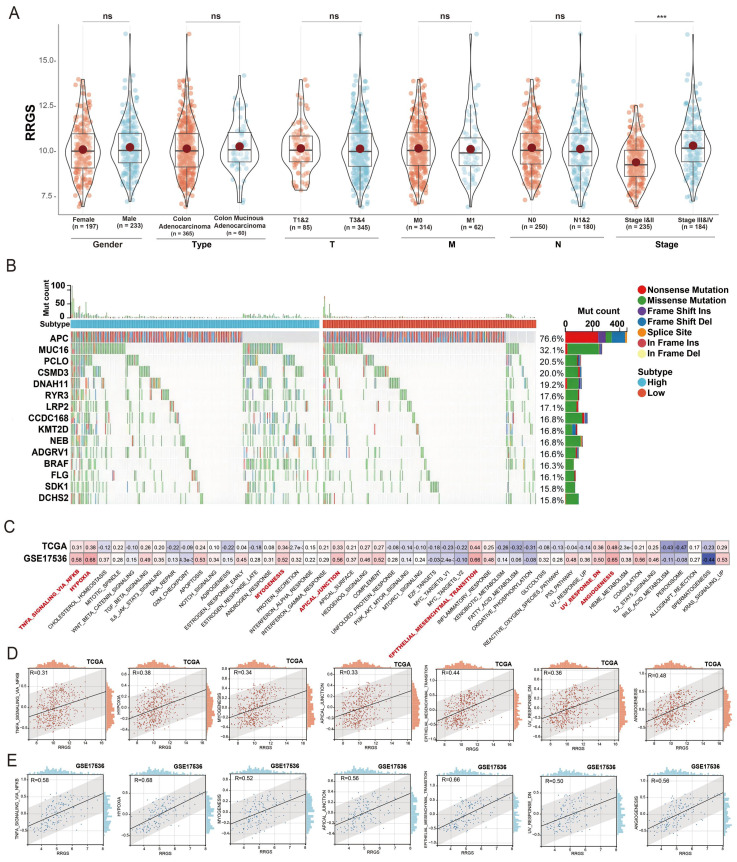
** Correlation analysis of risk score with clinical characteristics of colorectal cancer.** (A) Analysis of RRGS expression differences based on gender, tumor type, TMN staging, and overall stage using TCGA cohort; (B) Gene mutation analysis in RRGS-High and RRGS-Low subgroups; (C) GSVA analysis of the correlation between RRGS and different signaling pathways; (D) Correlation analysis of seven signaling pathways positively associated with RRGS in the TCGA cohort; (E) Correlation analysis of seven signaling pathways positively associated with RRGS in the GSE17536 cohort. NS, p>0.05; ***, p<0.001.

We further explored the relationship between the RRGS and clinical characteristics using the TCGA dataset. Our analysis indicated that RRGS values were not associated with patient gender, cancer type, or TNM; however, a significant association was observed with the overall cancer stage. Specifically, RRGS values were notably elevated in patients with Stage II and IV cancers compared to those with Stage I and III (Figure 4A).

Additionally, Figure 4B illustrates the distinct patterns of gene mutation rates between the RRGS-Low and RRGS-High subtypes. The RRGS-Low subgroup exhibited a higher mutation rate in the APC gene, while the RRGS-High subgroup presented increased mutation rates in genes such as MUC16, PCLO, CSMD3, DNAH11, RYR3, LRP2, CCDC168, KMT2D, NEB, ADGRV1, BRAF, FLG, SDK1, and DCHS2.

To unravel the mechanisms by which RRGS may contribute to colorectal cancer progression, we employed Gene Set Variation Analysis (GSVA) on the TCGA-KIRC and GSE17536 cohorts. Figure 4C details the correlation between RRGS and various signaling pathways across both cohorts, emphasizing significant associations with pathways such as TNFA signaling via NF-kB, hypoxia, myogenesis, formation of apical junctions, epithelial-mesenchymal transition (EMT), downregulation of UV response, and angiogenesis. Figure 4D elaborates these correlations within the TCGA dataset, whereas Figure 4E presents parallel analyses in the GSE17536 dataset.

### Assessment of RRGS and tumor immune microenvironment

**Figure 5:**
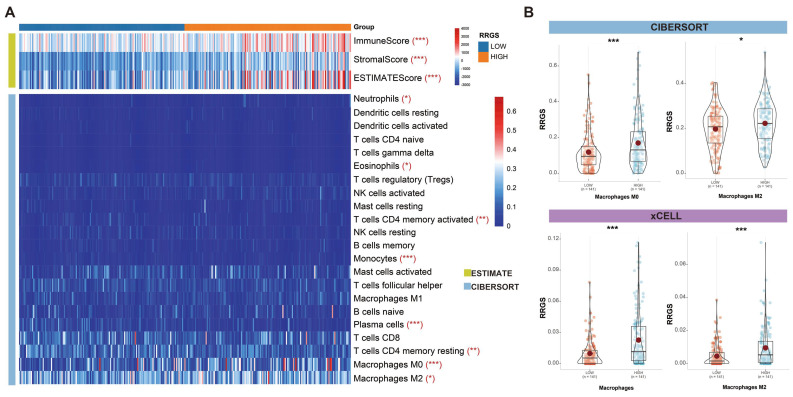
** Relationship between RRGS and the immune microenvironment.** (A) Results of differential immune cell infiltration between RRGS-High and RRGS-Low subgroups assessed by CIBERSORT and ESTIMATE; (B) The relative cell abundances of macrophages between the two groups are calculated using xCELL and CIBERSORT. *, p<0.05; ***, p<0.001.

To evaluate the capability of the RRGS in reflecting the tumor immune microenvironment, we estimated immune cell infiltration in colorectal cancer using three independent algorithms: ESTIMATE, CIBERSORT, and xCELL. Figure 5A illustrates the differences in infiltration scores and immune cell types between the RRGS-Low and RRGS-High subgroups. Notably, the RRGS-High subgroup exhibited an increased level of immune cell infiltration.

Among the immune cells, there was a pronounced presence of M0 and M2 macrophages in the RRGS-High subgroup. A comparative analysis using CIBERSORT and xCELL confirmed a significant upregulation of both total macrophages and M2 macrophages in this group (Figures 5B). These findings highlight distinct characteristics of the tumor immune microenvironment associated with the RRGS-High subgroup.

### Correlation of RRGS with prognosis in colorectal cancer patients post-chemotherapy

**Figure 6:**
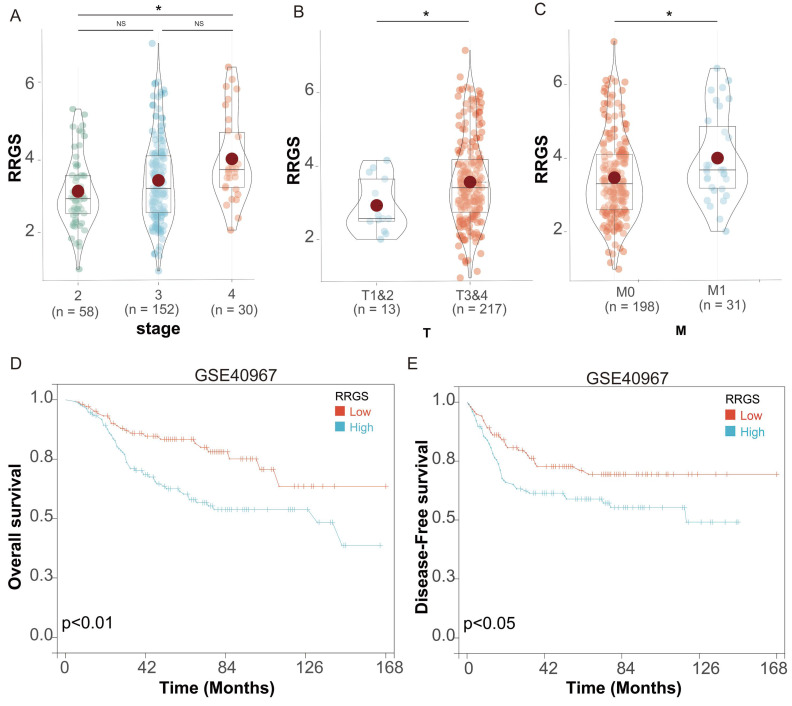
** Prognostic analysis of RRGS in colorectal cancer patients undergoing chemotherapy.** (A-C) Differential expression analysis of RRGS in colorectal cancer patients with chemotherapy from the GSE40967 dataset, based on (A) stage, (B) T classification, and (C) M classification; (D-E) The impact of RRGS on patients' overall survival (OS) and disease-free survival (DFS) in the GSE40967 cohort. NS, p>0.05; *, p<0.05.

We investigated the relationship between the RRGS and the prognosis of colorectal cancer patients following chemotherapy, utilizing data from the GSE40967 dataset. This dataset encompasses information on 240 patients who underwent chemotherapy and have subsequent follow-up data available for prognostic analysis. Our assessment of clinical characteristics demonstrated that RRGS values increased progressively with more advanced tumor stages within this dataset (Figure 6A). Specifically, RRGS values were significantly higher in stages T3&4 compared to T1&2 (Figure 6B), and in M1 compared to M0 stages (Figure 6C).

Survival analysis revealed that patients in the RRGS-Low subgroup exhibited significantly better OS than those in the RRGS-High subgroup (Figure 6D). Similarly, DFS was markedly improved in the RRGS-Low subgroup compared to the RRGS-High subgroup (Figure 6E).

### High expression of STC1 in colorectal cancer tissue is associated with poor prognosis

Given STC1's prominent contribution to the diagnostic model as evidenced by its highest coefficient, we specifically chose to investigate STC1 for functional studies. Its biological relevance is underscored by its role as a secreted glycoprotein involved in various processes, such as cell proliferation, angiogenesis, and tumor progression, making it a compelling candidate for deeper exploration in the context of colorectal cancer.

**Figure 7:**
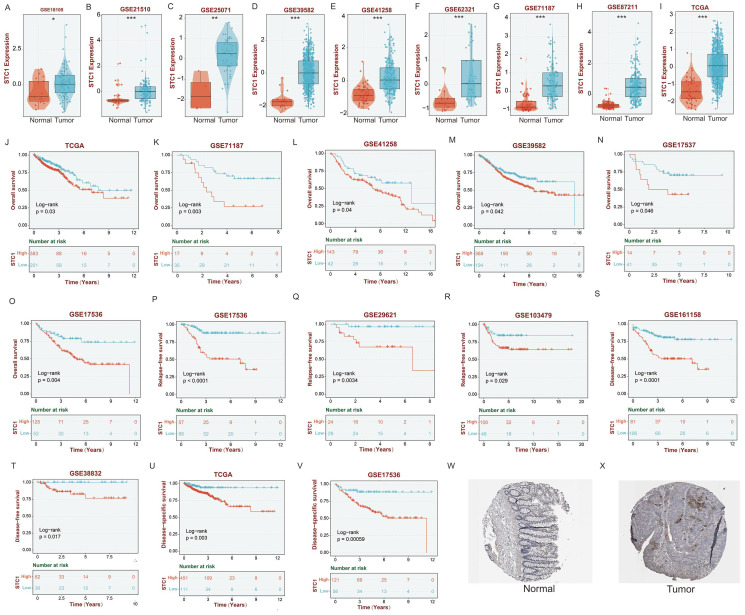
** High expression of STC1 in colorectal cancer tissue is associated with poor prognosis.** (A-I) The expression of STC1 between cancer tissues and adjacent non-cancerous tissues in (A) GSE18105, (B) GSE21510, (C) GSE25071, (D) GSE39582, (E) GSE41258, (F) GSE62321, (G) GSE71187, (H) GSE87211, and (I) TCGA cohort. (J-O) OS analysis of STC1 in the (J) TCGA, (K) GSE71187, (L) GSE41258, (M) GSE39582, (N) GSE17537, and (O) GSE17536 cohort. (P-R) RFS analysis in the (P) GSE17536, (Q) GSE29621, and (R) GSE103479 cohort. (S-V) DFS analysis of STC1 in the (S) GSE161158, (T) GSE38832, (U) TCGA, and (V) GSE17536 cohort. (W-X) STC1 protein expression in (W) colorectal cancer tissues and (X) adjacent normal tissues from the THPA database. *, p<0.05; **, p<0.01; ***, p<0.001.

Initially, we analyzed the expression levels of STC1 (Stanniocalcin 1) in colorectal cancer tissues. The results revealed that in the datasets GSE18105, GSE21510, GSE25071, GSE39582, GSE41258, GSE62321, GSE71187, GSE87211, and TCGA, STC1 was significantly overexpressed in cancer tissues compared to adjacent non-cancerous tissues (Figures 7A-I).

OS analysis demonstrated that in the TCGA, GSE71187, GSE41258, GSE39582, GSE17537, and GSE17536 datasets, patients with low STC1 expression had significantly better prognoses than those with high STC1 expression (Figures 7J-O). Similarly, relapse-free survival (RFS) analysis in the GSE17536, GSE29621, and GSE103479 datasets showed that patients with lower STC1 expression had markedly better outcomes (Figures 7P-R). Disease-free survival (DFS) analysis further supported these findings in the GSE161158, GSE38832, TCGA, and GSE17536 datasets, where low STC1 expression was associated with improved prognosis (Figures 7S-V).

Additionally, analyses of STC1 protein expression from the THPA database indicated that STC1 protein levels were significantly higher in colorectal cancer tissues compared to adjacent normal tissues (Figures 7W-X).

### Knockdown of STC1 inhibits proliferation and metastasis of colorectal cancer cells *in vitro* and *in vivo*

**Figure 8:**
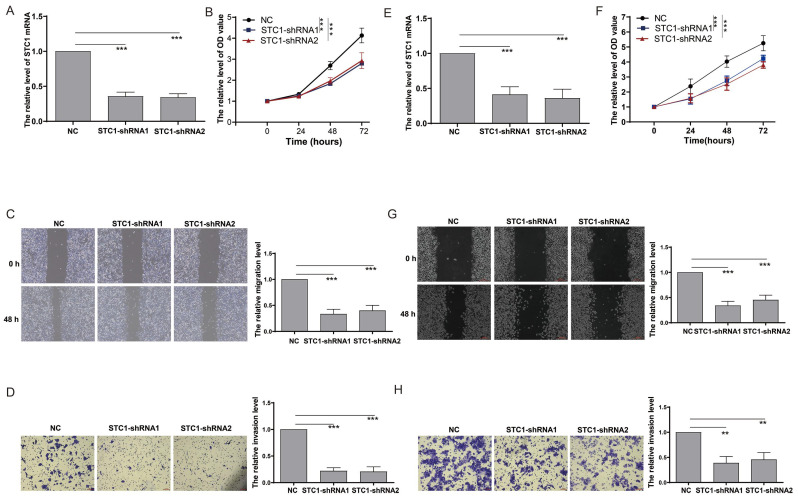
** Knockdown of STC1 inhibits the *in vitro* proliferation and metastasis capabilities of colorectal cancer cells.** (A) RT-PCR detection of STC1 expression after knockdown in HCT-116 cells; (B) CCK8 assay for changes in proliferation ability after STC1 knockdown in HCT-116 cells; (C-D) Effect of STC1 knockdown on migration and invasion abilities of HCT-116 cells (C) migration and (D) invasion; (E) RT-PCR detection of STC1 expression after knockdown in DLD1 cells; (F) CCK8 assay for changes in proliferation ability after STC1 knockdown in DLD1 cells; (G-H) Effect of STC1 knockdown on migration and invasion abilities of DLD1 cells (G) migration and (H) invasion.**, p<0.01; ***, p<0.001.

To elucidate the impact of STC1 on colorectal cancer cell functionality, we performed gene knockdown experiments in HCT-116 cells (Figure 8A). The CCK8 assay demonstrated that STC1 knockdown significantly reduced the proliferation rate of HCT-116 cells (Figure 8B). In parallel, the scratch assay revealed a marked suppression of cell migration following STC1 knockdown (Figure 8C). Furthermore, Transwell assay results confirmed a substantial inhibition of cell invasion capability in STC1-silenced HCT-116 cells (Figure 8D). Consistent results were observed in DLD1 colorectal cancer cells, where STC1 knockdown led to notable inhibition of cell proliferation, migration, and invasion (Figure 8E-H).

**Figure 9:**
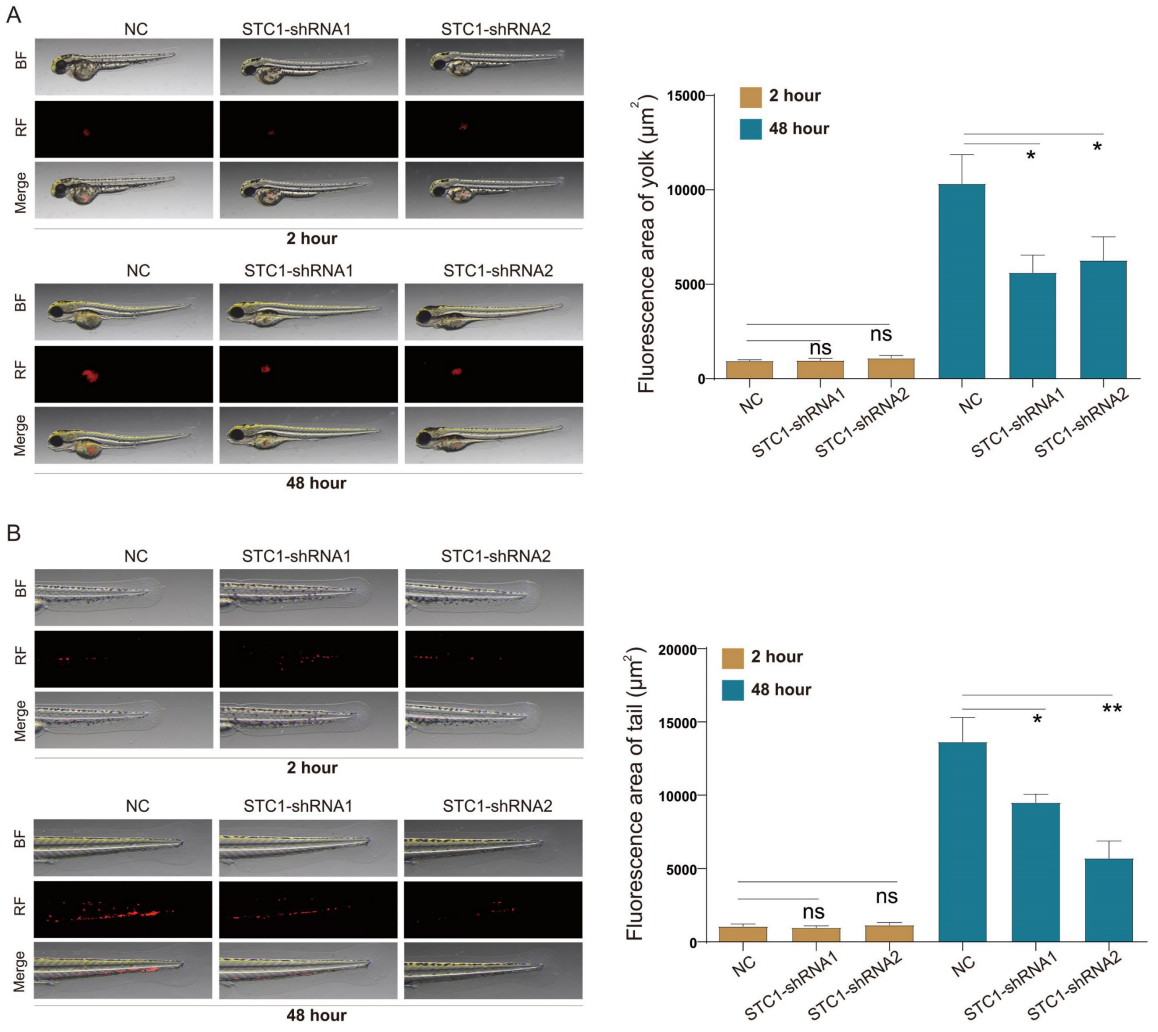
** Knockdown of STC1 inhibits the proliferation and metastasis capabilities of colorectal cancer cells in zebrafish.** Effect of STC1 knockdown on *in vivo* proliferation and metastasis capabilities of HCT-116 cells in zebrafish (A) proliferation and (B) metastasis. NS, p>0.05; *, p<0.05; **, p<0.01.

Leveraging the zebrafish model-an established vertebrate system whose signal transduction pathways and biological structures closely mirror those of humans-we investigated the *in vivo* effects of STC1 knockdown on HCT-116 cells. The transparency of zebrafish embryos allowed for real-time observation of tumor cell behavior. Our *in vivo* experiments demonstrated that STC1 knockdown notably impaired both the proliferation and metastasis of HCT-116 cells within zebrafish models (Figure 9A-B).

## Discussion

The importance of studying colorectal cancer recurrence cannot be overstated. Despite advancements in initial treatment strategies, including surgery, chemotherapy, and targeted therapies, a significant proportion of patients still experience disease recurrence, often with limited treatment options and poor prognosis^25^-^27^. Recurrence not only impacts patient survival but also places a substantial burden on healthcare systems and diminishes quality of life^28^. By focusing on the genetic underpinnings of recurrence, our study contributes to the body of knowledge on molecular biomarkers for colorectal cancer and provides new insights into the genetic basis of disease relapse.

Our identification of 79 DEGs between recurrent and non-recurrent colorectal cancer tissues provides a comprehensive view of the genetic alterations associated with recurrence. The significant enrichment of upregulated genes in recurrent tissues with cancer progression-related pathways such as focal adhesion, ECM-receptor interaction, and EMT aligns with current understanding of the molecular mechanisms driving cancer progression and metastasis. These pathways play crucial roles in cell migration, invasion, and adaptation to new microenvironments, all of which are essential for cancer recurrence and metastatic spread^29^-^31^. Conversely, the downregulation of immune-related genes in recurrent tissues suggests potential immune evasion mechanisms in recurrent tumors. This finding underscores the complex interplay between tumor cells and the immune microenvironment, highlighting the need for further investigation into immunotherapeutic approaches to prevent colorectal cancer recurrence.

The RRGS model constructed using LASSO regression demonstrated robust performance in distinguishing recurrent from non-recurrent CRC, with an AUC of 0.87. The model's ability to not only differentiate between recurrent and non-recurrent cases but also provide prognostic information regarding OS and DFS emphasizes its potential clinical utility. Patients with higher RRGS scores exhibited significantly poorer survival outcomes, underscoring the prognostic value of RRGS in clinical settings. The prognostic value of RRGS in patients who have undergone chemotherapy is a significant finding. The ability to predict outcomes post-chemotherapy can guide decisions on adjuvant treatments and follow-up regimens, potentially improving long-term survival rates. The correlation of RRGS scores with tumor progression stages, particularly stages II and IV, provides valuable prognostic information. This association suggests that RRGS could be used to identify patients at higher risk of recurrence even in early stages of the disease, potentially guiding more aggressive treatment strategies for these high-risk individuals. The strong correlation of RRGS with pathways such as TNFA signaling via NF-kB, hypoxia, and EMT further supports the biological relevance of our gene signature. These pathways are recognized contributors to cancer progression and metastasis, reinforcing the mechanistic basis of our model.

The distinct mutation spectra observed between the RRGS-Low and RRGS-High subtypes provide valuable insights into the genetic heterogeneity of colorectal cancer. In the RRGS-Low subgroup, the higher mutation rate of the APC gene is often associated with reduced tumor aggressiveness and a more favorable prognosis^32^. APC is a well-known tumor suppressor gene frequently mutated in colorectal cancer, with such mutations typically linked to early tumor development. In contrast, the RRGS-High subgroup is characterized by mutations in genes such as MUC16, PCLO, and CSMD3, which are commonly associated with more aggressive tumor features and poorer outcomes. The elevated mutation rates of these genes may contribute to increased tumor progression and metastatic potential. These findings suggest distinct evolutionary pathways and potential therapeutic targets for each of these subgroups.

Our analysis of the tumor immune microenvironment revealed higher levels of immune cell infiltration in the RRGS-High subgroup, particularly a marked upregulation of M0 and M2 macrophages. This finding is especially noteworthy as M2 macrophages are typically associated with immunosuppression, tissue repair, and tumor progression. The increased presence of these cells in high-risk tumors suggests a potentially strong immunosuppressive environment and a mechanism for recurrence. High expression levels of M2 macrophages in the RRGS-High subgroup may indicate increased tumor aggressiveness and a potential capacity for immune evasion. This suggests that tumors classified in the RRGS-High subgroup could exhibit enhanced aggressiveness and ability to evade the immune response.

Our comprehensive investigation of STC1, a key component of the RRGS, provides compelling evidence of its significant role in colorectal cancer progression. Originally discovered in fish as a glycoprotein hormone, STC1 is primarily involved in the regulation of calcium and phosphate metabolism. Recent studies in mammals have elucidated its complex roles in various physiological and pathological processes, including cancer^33^. In breast cancer, abnormal STC1 expression is closely tied to tumor growth and metastasis. It is suggested that STC1 exhibits oncogenic properties, potentially promoting tumor progression by stimulating cell proliferation and inhibiting apoptosis^34^. In melanoma, STC1 drives tumor progression by competitively binding to betaPIX, leading to the nuclear translocation of YAP and recruitment of M2 macrophages. This forms a YAP/CCL2/VEGFA/AKT feedback loop, which increases PD-L1 expression and enhances immune evasion^35^. Meanwhile, in gastric cancer, STC1 fosters tumor angiogenesis through the upregulation of VEGF, indicating its role in supporting tumor growth and metastasis by promoting new blood vessel formation^36^.

In colorectal cancer, recent research has shown that STC1 can enhance immune evasion and inhibit immune recognition. By upregulating PD-L1 expression, STC1 aids colorectal cancer cells in escaping immune system attacks, thereby facilitating tumor growth and progression^37^. However, detailed insights into STC1's impact on the functional behavior of colorectal cancer cells remain unexplored.

The consistent overexpression of STC1 across cancer tissues, as observed in multiple datasets, combined with its association with poor prognosis, underscores its potential as both a biomarker and a therapeutic target. The observed reduction in proliferation and metastasis following STC1 knockdown *in vitro* and *in vivo* further substantiates this potential. Our study notably utilizes zebrafish models, which provide distinct benefits in cancer research, such as rapid development, optical transparency for real-time imaging, and genetic similarity to humans. Our findings of reduced proliferation and metastasis in STC1-knockdown HCT-116 cells in zebrafish embryos offer a valuable *in vivo* validation of our *in vitro* results. This approach effectively bridges the gap between cell culture studies and mammalian models, offering a cost-effective and ethically favorable alternative for initial *in vivo* testing.

While our study provides valuable insights into the role of STC1 and the RRGS model in colorectal cancer recurrence, several limitations should be acknowledged. Firstly, our findings are primarily based on retrospective analyses of public datasets, which may introduce potential biases. Prospective clinical studies are needed to validate the prognostic value of RRGS and STC1 expression. Secondly, although we demonstrated the effects of STC1 knockdown *in vitro* and in zebrafish models, further investigations using mammalian models are necessary to fully elucidate its role in colorectal cancer progression. Thirdly, while we identified potential mechanisms through which STC1 may influence cancer progression, more detailed molecular studies are required to unravel the precise pathways involved. Future studies should aim to address these limitations and explore the potential of STC1 as a therapeutic target in colorectal cancer.

## Conclusion

In conclusion, our study presents a novel gene signature for predicting colorectal cancer recurrence, demonstrating robust diagnostic and prognostic capabilities. RRGS not only provides a risk stratification tool but also offers insights into the biological processes driving recurrence. The identification of STC1 as a crucial player in colorectal cancer progression opens new avenues for targeted therapies. Future research should focus on prospective validation of RRGS in larger and more diverse cohorts and explore its potential in guiding personalized treatment strategies. Furthermore, investigating the functional roles of genes in our signature, particularly STC1, may lead to new therapeutic approaches for preventing and managing colorectal cancer recurrence.

## Abbreviations

ADGRV1: Adhesion G Protein-Coupled Receptor V1
AKAP12: A-Kinase Anchoring Protein 12
APC: Adenomatous Polyposis Coli
BRAF: B-Raf Proto-Oncogene, Serine/Threonine Kinase
CAV2: Caveolin 2
CCL11: C-C Motif Chemokine Ligand 11
CCDC168: Coiled-Coil Domain Containing 168
CPE: Carboxypeptidase E
CSMD3: CUB And Sushi Multiple Domains 3
DCHS2: Dachsous Cadherin-Related 2
DNAH11: Dynein Axonemal Heavy Chain 11
FAM3B: Family With Sequence Similarity 3 Member B
FLG: Filaggrin
KMT2D: Lysine Methyltransferase 2D
L1TD1: LINE-1 Type Transposase Domain Containing 1
LRP2: LDL Receptor Related Protein 2
MAGEA6: MAGE Family Member A6
MMP3: Matrix Metallopeptidase 3
MUC16: Mucin 16, Cell Surface Associated
NEB: Nebulin
PCLO: Piccolo Presynaptic Cytomatrix Protein
RYR3: Ryanodine Receptor 3
SDK1: Sidekick Cell Adhesion Molecule 1
SERPINE1: Serpin Family E Member 1
SFRP2: Secreted Frizzled Related Protein 2
STC1: Stanniocalcin 1
